# The impacts of COVID‐19 measures on drug markets and drug use among a cohort of people who use methamphetamine in Victoria, Australia

**DOI:** 10.1111/add.16189

**Published:** 2023-04-02

**Authors:** Kasun Rathnayake, Paul A. Agius, Bernadette Ward, Matthew Hickman, Lisa Maher, Mark Stoové, Joseph S. Doyle, Margaret Hellard, Anna Wilkinson, Brendan Quinn, Sione Crawford, Keith Sutton, Paul Dietze

**Affiliations:** ^1^ Burnet Institute Melbourne Victoria Australia; ^2^ Faculty of Health Deakin University Melbourne Victoria Australia; ^3^ School of Public Health and Preventive Medicine Monash University Melbourne Victoria Australia; ^4^ Melbourne School of Population and Global Health University of Melbourne Melbourne Victoria Australia; ^5^ School of Rural Health Monash University Bendigo Victoria Australia; ^6^ Population Health Sciences, Bristol Medical School University of Bristol Bristol UK; ^7^ Kirby Institute University of New South Wales Sydney New South Wales Australia; ^8^ Department of Infectious Diseases The Alfred Hospital Melbourne Victoria Australia; ^9^ Doherty Institute University of Melbourne Melbourne Victoria Australia; ^10^ Australian Institute of Family Studies Melbourne Victoria Australia; ^11^ Harm Reduction Victoria Melbourne Victoria Australia; ^12^ National Drug Research Institute Melbourne Office Curtin University Melbourne Victoria Australia

**Keywords:** Australia, COVID‐19, data, illicit drugs, longitudinal, methamphetamine, people who use drugs, public health measures

## Abstract

**Background and Aims:**

Few studies of the impacts of the coronavirus disease 2019 (COVID‐19) public health measures on drug markets and drug use patterns have used longitudinal data. We aimed to examine whether COVID‐19 measures were associated with increases in methamphetamine price, decreases in methamphetamine use frequency and subsequent changes in secondary outcomes of other drug use frequency in metropolitan Melbourne and regional Victoria.

**Design:**

Longitudinal analysis framework was used from a longitudinal cohort of people who use methamphetamine.

**Setting:**

Victoria state, Australia.

**Participants:**

One hundred eighty‐five VMAX study participants who reported a methamphetamine purchase after the onset of the pandemic were used for the price paid analysis. Methamphetamine or other drug use frequency analysis was performed using 277 participants who used methamphetamine during the pandemic or in the year before the pandemic.

**Measurements:**

Price paid per gram of methamphetamine derived from the most recent purchase price and most recent purchase quantity. Frequency of methamphetamine and other drug use measured as the average number of days per week used in the last month.

**Findings:**

Compared with pre‐COVID‐19 period, methamphetamine prices increased by AUD351.63 (*P* value <0.001) and by AUD456.51 (*P* value <0.001) in Melbourne and regional Victoria, respectively, during the period in which the most intense public health measures were implemented in Victoria. Although prices decreased after harder restrictions were lifted (by AUD232.84, *P* value <0.001 and AUD263.68, *P* value <0.001, in Melbourne and regional Victoria, respectively), they remained higher than pre‐COVID‐19 levels. A complementary 76% decrease was observed in relation to methamphetamine use frequency in regional Victoria (*P* value = 0.006) that was not offset by any changes in the frequency of use of other drugs such as alcohol, tobacco or other illicit drugs.

**Conclusion:**

COVID‐19 public health measures in Victoria state, Australia, appear to have been associated with major price changes in the methamphetamine market and decreased frequency of use of the drug.

## INTRODUCTION

Many governments implemented public health measures to stem severe acute respiratory syndrome coronavirus 2 (SARS‐CoV‐2) transmission, including border closures and other restrictions on movement (e.g. ‘lockdowns’). Frontline workers, researchers and other experts reported their perceptions of the impacts of these measures and associated changes in service delivery on drug use, drug markets and drug‐related harms [1, 2, 3, 4, 5]—perceptions that can now be tested using surveillance and other datasets that are now available [6, 7].

Many studies of coronavirus disease 2019 (COVID‐19) measures’ impacts on drug use have relied on surveillance datasets such as hospitalisations, wastewater, ambulance attendances or mortality [6, 8, 9, 10]. Trends in these datasets can be difficult to interpret because COVID‐19 related measures such as lockdowns may have induced other changes in market parameters such as the prevalence and frequency of use of particular substances and their availability, quality and price, which can all influence drug‐related harms [11]. Most direct assessments of COVID‐19’s impacts on people who use drugs are hampered by samples recruited after the onset of the pandemic and reliance on questions that are susceptible to recall and social desirability biases. Surveillance studies of people who use drugs rely on serial cross‐sectional sampling, which can be affected by changes in methodology, as well as sample composition, during the pandemic [7].

In Australia, the first case of COVID‐19 was detected in January 2020; with case numbers rising, public health measures were introduced in March, including a nationwide lockdown involving international and domestic border closures and restrictions on non‐essential gatherings and movement imposed by state, territory and federal governments, designed to ‘flatten the curve’ of infections [1, 12]. Initial public health measures proved largely successful and began to lift in mid‐2020 [7], but following an outbreak from hotel quarantine facilities in Melbourne, more restrictions and lockdown measures were introduced in the state of Victoria. In metropolitan Melbourne (‘Melbourne’), restrictions on movement were much stricter (e.g. a curfew, few permitted reasons for leaving home and time‐limited exercise within a five kilometre boundary) than the rest of the state, and borders with adjacent states (South Australia and New South Wales) were closed from July 2020 until November 2020 [13]. Although lockdown measures were lifted in late 2020 [13], re‐emergence of outbreaks before vaccination became widespread saw Melbourne enter and leave lockdown conditions such that, by late 2021, it was often described as the ‘most locked‐down city in the world’ [14].

Previous research has highlighted how key drug‐related parameters such as price are linked to the risks that people who distribute and consume drugs face, with increased risks thought to increase drug prices [15, 16, 17]. Consistent with this ‘risks and prices’ framework, it was expected that border closures would interrupt drug supply and restrictions on movement and curfews would increase risks to distributors and consumers [1], thereby increasing prices and affecting consumer behaviours. Initial evidence suggested that people who inject drugs in Melbourne were at increased risk of enforcement of lockdown measures [18] and that Melbourne’s lockdowns increased prices paid for key drugs (heroin and methamphetamine), but had little effect on availability or purity [19].

Although prospective cohort studies are suited to monitoring the effects of naturally occurring ‘big events’ [20] such as the COVID‐19 pandemic, few such studies of COVID‐19 impact on drug markets and drug use have occurred. VMAX is a prospective cohort study of people who use methamphetamine recruited from Melbourne and regional Victoria. Established in 2016, VMAX collects information on patterns of drug use over time as well as information related to key drug market parameters such as price, quantity and use frequency. Participant interviews continued after the pandemic began and throughout lockdown phases, enabling examination of within‐participant impacts of the pandemic. We used longitudinal data from VMAX from June 2016 to May 2021 to explore:

- the effects of the key phases of the Victorian pandemic response mainly on methamphetamine price and methamphetamine use frequency and how these varied according to a range of exposures, in particular Melbourne or regional Victorian residence, and
- the changes in reports of the use frequency of other drugs related to the pandemic restrictions, which can be used to explain the changes of methamphetamine use frequency.

Consistent with the risks and prices framework outlined above, we hypothesised that restrictions on movement limited drug market operations, increasing risks for suppliers and causing higher prices. We expected that use frequency would decline, potentially because of higher prices, but also because of restrictions on movement limiting drug market access.

## METHODS

### Study design

We used a longitudinal analysis framework to primarily examine the impacts of COVID‐19 restrictions in Victoria on reports of methamphetamine price and methamphetamine use frequency among the VMAX cohort. Three secondary outcomes were examined including use frequency of tobacco, alcohol and other illicit drugs.

VMAX data have been collected via face‐to‐face and telephone interviews with 853 participants recruited before the pandemic began. Eligibility criteria for the study were being aged ≥18 years, at least monthly use of any form of methamphetamine in the last six months, residing in one of the four recruitment locations (Melbourne or one of three regional locations in Victoria) and primarily using methamphetamine via non‐injecting routes (e.g. smoking and snorting). To reach the target sample size, the route of administration criterion was relaxed eventually to allow inclusion of injecting as a primary route of administration. During interviews, questionnaires are administered that cover domains including drug use, drug purchases and demographics. Participants are followed up annually with a modified version of the original baseline questionnaire and a shortened version in the intervening six months.

Preliminary data cleaning was performed on the interview records and the cleaning process is outlined in the results (Section 3). We then undertook the following steps to extract the study cohort for each analysis.

1. First, to select the samples to study the two primary outcome measures, methamphetamine price and methamphetamine use frequency, an intermediate sample was extracted from the pre‐cleaned data for participants interviewed at least once after pandemic onset.
2. Second, to examine methamphetamine price, interview records were used from participants who reported at least one methamphetamine purchase since 16 March 2020 and a recorded past‐month methamphetamine use in the same interview.
3. Finally, to examine methamphetamine use frequencies, a sample comprised of combined data from participants who reported methamphetamine use during the pandemic and those who did not report use during the pandemic, but did so in the previous 12 months before the pandemic was extracted. The purpose of including participants who did not report use during the pandemic was to include those who switched their drug choice from methamphetamine to another during the pandemic. This sample was used for analysis of methamphetamine use frequency, along with secondary outcomes of the use frequency of other drugs. A sensitivity analysis was conducted on these drug use frequencies, restricted to those who reported methamphetamine use after pandemic onset.

VMAX procedures are approved by The Alfred Hospital (171/16) and Monash University Human Research Ethics Committees (2938).

### Measures

#### Primary outcomes

This study involved two primary outcome measures.

1. Methamphetamine price, normalised as price paid per gram, was calculated as a ratio of:
  - purchase price: participants were asked to self‐report the amount paid (AUD) for the number of grams purchased; and
  - purchase quantity: participants were asked about the number of grams acquired in their most recent purchase.
2. Methamphetamine use frequency: at biannual follow‐ups, if participants indicated they had used methamphetamine in the month preceding interview, they were asked to estimate the average number of days per week methamphetamine was used in the past month. This average frequency (in days) was summed across all forms: powder, crystalline, base and liquid methamphetamine.

#### Secondary outcomes

This study examined three secondary outcomes. Participants were asked about their use of tobacco, alcohol and other illicit drugs in the past month separately. Illicit drugs included non‐prescribed pharmaceutical stimulants, ecstasy/3,4‐Methyl enedioxy methamphetamine (MDMA), cocaine, gamma‐Hydroxybutyric (GHB)/gamma butyrolactone (GBL)/1,4‐butanediol (1,4‐B), ketamine, benzodiazepines, heroin, hallucinogens, non‐prescribed pharmacotherapy, amyl nitrate/poppers, cannabis/pot/weed/marijuana, illicit antipsychotics and illicit pregabalin. Use frequency of these other illicit drugs was obtained by summing across the drugs listed above, and these three outcomes were measured as the average number of days per week used in the past month.

#### Main exposure

**FIGURE 1:**
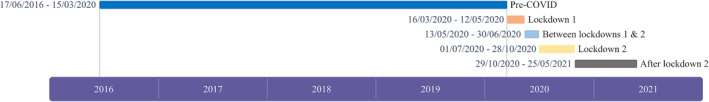
Timeline of the coronavirus disease 2019 (COVID‐19) restriction periods.

A time‐varying factor, COVID‐19 restriction periods (pre‐COVID‐19 [reference category], Lockdown 1, between Lockdowns 1 and 2, Lockdown 2 and after Lockdown 2) were considered as the main exposure and shown in Figure 1. These restriction periods were based on the start and end dates of the lockdown periods defined by the Victorian state government. To minimise the effects from overlapping restriction periods during the interviews, a 14‐day lag window was used for each restriction period (e.g. for the restriction period ‘during Lockdown 1’, which lasted from 16 March 2020 to 12 May 2020, interviews conducted between 30 March 2020 and 26 May 2020 have been considered). Further details of COVID‐19 restrictions are provided in Supporting information Table S1.

#### Covariates

Participant age (years) and gender (male vs female) at baseline were included as time‐invariant factors. Employment status (employed vs unemployed), accommodation (stable vs unstable), recruitment location (Melbourne vs regional Victoria) and average weekly income (below AUD600 vs above) were measured at each interview and included as time‐varying factors. Time in the study (years since enrolment) was measured as a time‐independent adjustment to COVID‐19 restriction periods.

- Drug source status, where participants were asked about the source of their last methamphetamine purchase, dichotomised into known versus unknown was included to identify whether time‐varying changes in sourcing were related to lockdown restrictions. Drug treatment status, where participants were asked whether they had engaged in any drug treatment programmes for methamphetamine use in the previous 12 months was also included as a time‐varying exposure.

A detailed description of these covariates is given in Supporting information Table S2.

### Statistical analysis

First, we explored the relationship between COVID‐19 restriction periods on key outcome measures. Descriptive statistics were generated and compared between those included and excluded from the two analytic samples using a χ^2^ test for association for categorical variables and a two‐sample *t* test for continuous variables. Descriptive statistics by restriction periods and trajectories of the key outcome measures were plotted. A linear mixed effects model (LMM) was used to model methamphetamine price as it was continuous, measured repeatedly among participants. A Poisson generalised linear mixed effects model (GLMM) was used to explore use frequency because the measuring unit was average number of days of use. The second phase of the analysis involved investigating COVID‐19 restrictions’ effect on use frequency of alcohol, tobacco and other illicit drugs. Because of the excessive number of ‘real zero’ responses and after accounting for overdispersion, zero‐inflated Poisson GLMM was used to model alcohol use frequency and zero‐inflated negative binomial GLMM was used to model other illicit drug use frequency. Poisson GLMM was used to model changes of tobacco use frequency. Each model was clustered with two levels: level 1—participant response and level 2—participant. In these models age at baseline, gender, employment, income, accommodation, recruitment location, COVID‐19 restriction period and time in the study were estimated as fixed effects, and to account for the correlation between participant repeated measures, a random intercept for individuals was also estimated. To investigate the effect on two primary outcomes, in addition to the fixed effects listed above, drug source and drug treatment status were also estimated as fixed effects and an interaction term between recruitment location and restriction period was also estimated, reflecting the different restrictions operating in Melbourne and regional Victoria during the lockdown periods and the extent to which this contributed to location‐specific heterogeneity in COVID‐19 restriction effects. For the other illicit drugs model, a similar interaction term was included, because frequency of use of these drugs could vary as a function of the different restrictions in recruitment location; alcohol and tobacco were both readily available throughout Victoria during this time and so an interaction term was not included in these models. A sensitivity analysis that was conducted on drug use frequencies based on all participants who reported methamphetamine use during the pandemic is given in Supporting information Table S3.

A complete case approach was used to manage missing data. Records where methamphetamine price per gram <AUD10 or >AUD2000 were identified as outliers and excluded from the analysis. Model estimates for each of these models along with standard errors, confidence intervals and *P* values are given in the results section. In addition, effects sizes for key findings are presented in the Supporting Information (Tables S4 and S5). Cohen’s *D* values were calculated as a measure of effect sizes for all the models considered and the calculations were performed using *eff_size* function available in the R environment [21]. Statistical analyses were conducted using RStudio Version 1.4.1106, with a threshold of 0.05 (α) used to determine statistical significance. As the analysis was not pre‐registered, the results should be considered exploratory.

## RESULTS

**FIGURE 2:**
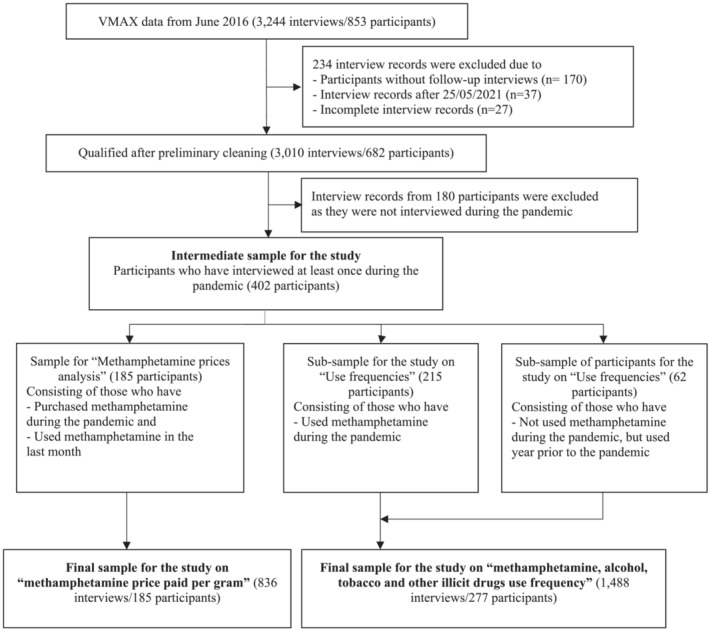
Flow chart for the study sample selections.

At 25 May 2021, VMAX had 3244 interview records, 2516 interviews were conducted before the onset of COVID‐19 (before 16 March 2020) and 728 interviews were conducted after the onset. We excluded 234 interview records from 171 participants (of 853 interviewed from 17 June 2016) during the data pre‐processing stage (Figure 2). Most interviews (77.04%) occurred during pre‐COVID‐19 period; the fewest occurred during Lockdown 1 and between Lockdowns 1 and 2 (3.75% and 3.12%, respectively), periods of only 2 months. After applying inclusion criteria, for studying methamphetamine price and use frequency, 836 and 1488 interview records were available, respectively.

### Sample characteristics

Table 1 shows the baseline characteristics for participants included in and excluded from analyses. In both study samples, most of the participants were male, the mean age at baseline was above 35 years, most reported being unemployed at baseline, over four fifths were living in stable accommodation and more than one quarter reported having engaged in drug treatment programme in the previous 12 months. Most of the participants were recruited from regional Victoria, and nearly 90% reported purchasing drugs from a known source at baseline. Baseline characteristics of the participants excluded from these analyses were similar, apart from being significantly younger and having a higher percentage recruited from regional Victoria.

**TABLE 1:** Baseline characteristics of included and excluded participants of the analysis samples^a^

	As used for prices paid for methamphetamine^b^			As used for use frequencies of methamphetamine^c^		
	*n* included (%)	*n* excluded (%)	*P* value	*n* included (%)	*n* excluded (%)	*P* value
Gender: male	112 (60.54)	301 (60.56)	0.996	174 (62.82)	239 (59.01)	0.318
Age at baseline (years), mean (SD)	36.65 (9.61)	34.04 (9.64)	**<0.001**	35.77 (9.72)	34.05 (9.61)	**0.023**
Employment status: employed	42 (22.70)	103 (20.72)	0.575	69 (24.91)	76 (18.77)	0.054
Income per week: above AUD600	47 (25.54)	114 (22.94)	0.511	69 (24.91)	92 (22.72)	0.537
Accommodation type: stable	151 (81.62)	442 (88.93)	**0.010**	234 (84.48)	359 (88.64)	0.094
Drug sourcing method: known	161 (87.03)	417 (83.90)	0.618	233 (84.12)	345 (85.19)	0.711
On a drug treatment programme in the past 12 months	54 (29.19)	145 (29.18)	0.997	76 (27.44)	123 (30.37)	0.408
Recruitment location: Melbourne	78 (42.16)	151 (30.38)	**0.004**	117 (42.24)	112 (27.65)	**<0.001**

*Note*: Statistical significances are provided in bold font.

^a^ χ^2^ tests were used for identifying associations between two categorical variables; the unpaired two‐sample *t* test was used for investigating between continuous/parametric variable and a categorical variable with two levels with means and SD reported. This summarised table is based on the sample obtained after performing preliminary cleaning (682 participants).

^b^ The cohort consisted of 185 participants included in and 497 participants excluded from the study.

^c^ The cohort consisted of 277 participants included in and 405 participants excluded from the study.

Measures for time‐varying exposures at each lockdown period for both study samples are detailed in Table 2, showing that the ‘income per week’ for both samples and ‘recruitment location’ for methamphetamine use frequency are significant. *Post hoc* analyses show that the percentage of the sample with weekly income over AUD600 was significantly higher during Lockdown 2 compared to the pre‐COVID‐19 period in the samples used for methamphetamine prices and use frequency analyses. Percentages were highest during Lockdown 2, but declined significantly thereafter for both study samples. Recruitment from Melbourne was significantly higher between Lockdowns 1 and 2 compared to the pre‐COVID‐19 period for the methamphetamine use frequency sample.

**TABLE 2:** Characteristics of the time‐varying exposures during each of the restriction periods for study samples.

	Pre‐COVID‐19	During Lockdown 1	Between Lockdowns 1 and 2	During Lockdown 2	After Lockdown 2	*P* value
Methamphetamine price paid analysis						
Employment status: employed, *n* (%)	132 (24.86)	11 (22.92)	10 (21.74)	28 (24.35)	18 (19.35)	0.829
Income per week: above AUD600, *n* (%)^a^	126 (23.95)	16 (32.65)	16 (34.78)	56 (48.70)	25 (27.17)	**<0.001**
Accommodation type: stable, *n* (%)	461 (86.49)	42 (85.71)	36 (78.26)	97 (84.35)	74 (80.43)	0.391
Drug sourcing method: known, *n* (%)	484 (93.26)	47 (97.92)	40 (88.89)	109 (97.78)	87 (96.67)	0.282
On a drug treatment programme in the past 12 months, *n* (%)	135 (25.33)	11 (22.45)	10 (21.74)	21 (18.26)	23 (24.73)	0.594
Recruitment location: Melbourne, *n* (%)	260 (48.78)	28 (57.14)	29(63.04)	58 (50.43)	45 (48.39)	0.338
Methamphetamine use frequency analysis						
Employment status: employed, *n* (%)	272 (27.39)	18 (23.38)	13 (20.97)	39 (24.22)	39 (23.78)	0.601
Income per week: above AUD600, *n* (%)^a^	250 (25.41)	25 (31.65)	20 (32.26)	82 (51.57)	49 (30.06)	**<0.001**
Accommodation type: stable, *n* (%)	872 (87.81)	72 (91.14)	51 (82.26)	138 (85.71)	138 (85.19)	0.454
Drug sourcing method: known, *n* (%)	726 (92.25)	60 (96.77)	44 (86.27)	121 (95.28)	104 (94.55)	0.148
On a drug treatment programme in the past 12 months, *n* (%)	226 (22.51)	16 (19.51)	12 (19.35)	26 (15.76)	31 (17.71)	0.241
Recruitment location: Melbourne, *n* (%)^a^	490 (48.80)	52 (63.41)	42(67.74)	88 (53.33)	94 (53.71)	**0.005**

*Note*: Statistical significances are provided in bold font.

Abbreviation: COVID‐19, coronavirus 2019.

^a^ *Post hoc* analysis was performed for significant characteristics observed and interpreted in the Section 3.1.

### Prices paid per gram for methamphetamine and methamphetamine use frequency

**FIGURE 3:**
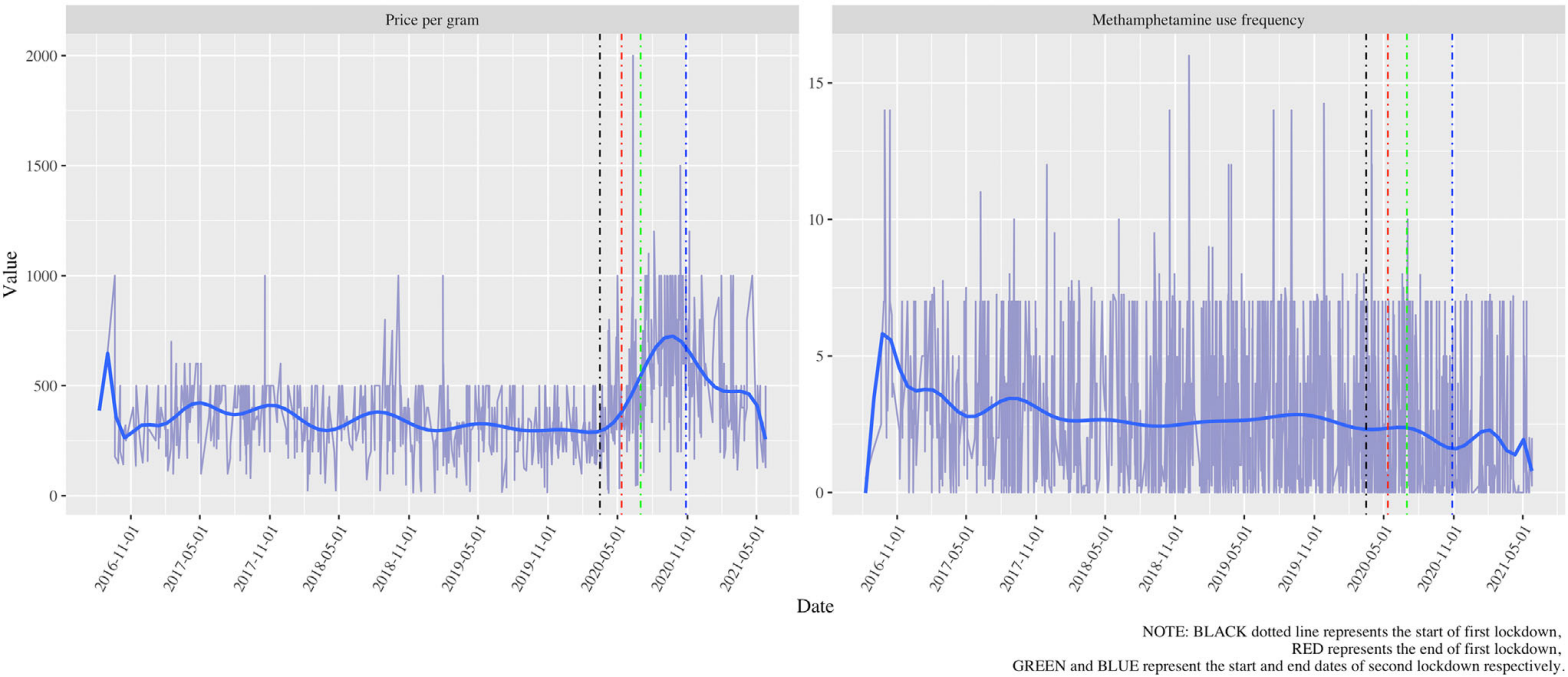
Trajectories of price per gram and methamphetamine use frequency over time.

Figure 3 and Table 3 show shifts in price paid per gram and methamphetamine use frequency across the interviews. Table 3 also shows the total number of interviews conducted in each period. Price paid per gram was relatively consistent during the pre‐COVID‐19 period, decreased slightly during Lockdown 1 and increased between Lockdowns 1 and 2, peaking in Lockdown 2 and declining after restrictions eased. Similarly, methamphetamine use frequency declined during Lockdown 1, during Lockdown 2 and after Lockdown 2 compared to the preceding period. Notably, the lowest average methamphetamine use frequency was recorded after Lockdown 2. Shifts in other drug use frequencies are also given in Table 3.

**TABLE 3:** Summary statistics of primary and secondary outcome measures.

	All	Pre‐COVID‐19	During Lockdown 1	Between Lockdowns 1 and 2	During Lockdown 2	After Lockdown 2
‘Methamphetamine price’ study: number of interviews (%)	836	533 (63.76)	49 (5.86)	46 (5.50)	115 (13.76)	93 (11.12)
‘Use frequency’ study: number of interviews (%)	1488	1004 (67.47)	82 (5.51)	62 (4.17)	165 (11.09)	175 (11.76)
Methamphetamine price per gram: mean (SD)	409 (226)	338 (148)	322 (193)	445 (290)	681 (267)	506 (231)
Methamphetamine use frequency: mean (SD)	2.58 (2.77)	2.84 (2.82)	2.06 (2.84)	2.41 (2.62)	2.06 (2.49)	1.87 (2.55)
Alcohol use frequency: mean (SD)	1.25 (2.10)	1.24 (2.09)	1.32 (2.05)	1.38 (2.10)	1.26 (2.16)	1.23 (2.13)
Tobacco use frequency: mean (SD)	4.85 (3.09)	4.83 (3.10)	5.01 (3.05)	5.76 (2.51)	4.87 (3.12)	4.53 (3.22)
Other illicit drugs use frequency: mean (SD)	3.03 (3.51)	3.08 (3.59)	3.09 (3.54)	3.68 (3.60)	3.17 (3.36)	2.29 (2.98)

**FIGURE 4:**
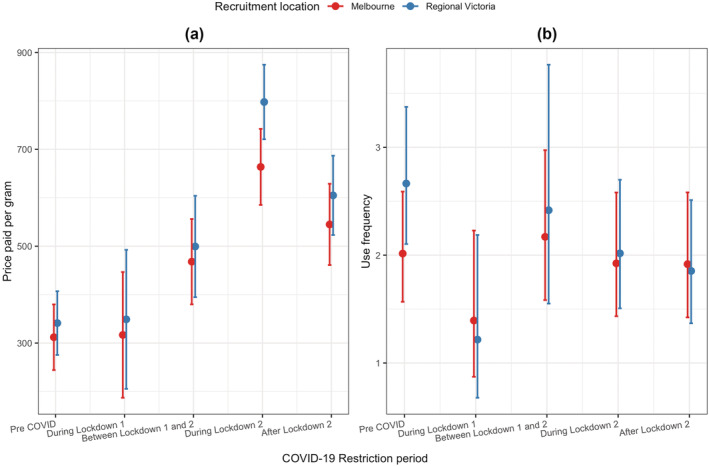
Marginal effects of interaction terms between coronavirus disease 2019 (COVID‐19) restriction period and recruitment location on methamphetamine (a) price per gram and (b) use frequency.

Table 4 shows the relationship between methamphetamine price and COVID‐19 restriction period, after adjusting for sociodemographic and methamphetamine consumption‐related measures. The model coefficients represent the estimated average change in price relative to covariates for methamphetamine price. The marginal effects of interactions between restriction period and recruitment location are plotted in Figure 4a and show that the prices in regional Victoria were higher compared to Melbourne. As indicated, the interaction effect between location and restriction period showed a clear tendency to significance (*χ* ^2^(4) = 8.98, *P* = 0.063), mainly driven by different effects of Lockdown 2 in the two recruitment locations. Compared to the pre‐COVID‐19 period, during Lockdown 2 methamphetamine prices increased by AUD351.63 (95% CI = 296.71–406.56, *P* < 0.001) and AUD456.51 (95% CI = 402.71–510.32, *P* < 0.001) per gram in Melbourne and regional Victoria, respectively. For both locations, the change in prices was significant compared to the pre‐COVID‐19 period except during Lockdown 1. Although prices fell in Melbourne and regional Victoria after Lockdown 2, they were still significantly higher than pre‐COVID‐19 measures by AUD232.84 (95% CI = 169.76–295.92, *P* < 0.001) and AUD263.68 (95% CI = 203.31–324.15, *P* < 0.001), respectively. Effect of location was significant only during Lockdown 2 and the prices in regional Victoria higher than Melbourne by AUD134.02 (95% CI = 67.16–200.88, *P* < 0.001). Time in the study was also significantly associated with price, with price reducing by AUD32 per year on average.

**TABLE 4:** Linear mixed effects model showing associations with methamphetamine price paid per gram: model estimates, standard error values and probability values (*P* values) (*n* = 185 participants).

Covariates	Price paid per gram		
	Estimate (SE)	CI	*P*
Age at baseline, years	1.61 (0.98)	−0.32 to 3.53	0.102
Male	−32.79 (18.22)	−68.56 to 2.98	0.072
Employed	20.19 (18.60)	−16.32 to 56.70	0.278
Income above AUD600 per week	−12.12 (16.65)	−44.80 to 20.56	0.467
Stable accommodation	−2.87 (18.56)	−39.30 to 33.56	0.877
Known drug source	8.52 (26.23)	−42.97 to 60.02	0.745
On a drug treatment programme in the past 12 months	14.55 (15.75)	−16.36 to 45.46	0.356
Time in the study	−32.27 (6.56)	−45.15 to −19.38	**<0.001**
Melbourne recruitment	−29.14 (19.16)	−66.75 to 8.47	0.129
COVID‐19 restriction period (Ref: Pre‐COVID‐19)			**<0.001**
During Lockdown 1	7.74 (67.58)	−124.91 to 140.40	0.909
Between Lockdowns 1 and 2	158.20 (45.87)	68.15 to 248.25	**0.001**
During Lockdown 2	456.51 (27.41)	402.71 to 510.32	**<0.001**
After Lockdown 2	263.68 (30.81)	203.21 to 324.15	**<0.001**
Interaction between COVID‐19 restriction period and recruitment location (Ref: Pre‐COVID‐19 and regional Victoria)			0.063
During Lockdown 1: Melbourne	−3.00 (89.18)	−178.06 to 172.05	0.973
Between Lockdowns 1 and 2: Melbourne	−2.22 (55.73)	−111.61 to 107.17	0.968
During Lockdown 2: Melbourne	−104.88 (35.40)	−174.37 to −35.39	**0.003**
After Lockdown 2: Melbourne	−30.84 (39.58)	−108.53 to 46.84	0.436
(Intercept)	395.55 (33.44)	329.91 to 461.18	**<0.001**

*Note*: Statistical significances are provided in bold font.

Abbreviations: COVID‐19, coronavirus 2019; Ref, reference category.

Relative risk ratios representing relative difference between incidence rates for the methamphetamine use frequency model are given in Table 5. There was a marginal trend toward a significant interaction effect between location and restriction period (*χ* ^2^(4) = 9.32, *P* = 0.054), driven largely by differential changes in the recruitment locations during the pandemic (Figure 4b). In regional Victoria, methamphetamine use frequencies during Lockdown 1, during Lockdown 2 and after Lockdown 2 were significantly lower than in the pre‐COVID‐19 period by a factor of 46% (95% CI = 0.27–0.79, *P* = 0.005), 76% (95% CI = 0.62–0.92, *P* = 0.006) and 70% (95% CI = 0.56–0.86, *P* < 0.001), respectively. Between Lockdowns 1 and 2, use frequencies were 56% (RR = 1.56, 95% CI = 0.98–2.46, *P* = 0.058) and 99% (RR = 1.99, 95% CI = 1.03–3.85, *P* = 0.042) higher than Lockdown 1 figures in Melbourne and regional Victoria, respectively. The effect of location was significant only during the pre‐COVID‐19 period and the use frequencies in Melbourne lower than regional Victoria by 24% (RR = 0.76, 95% CI = 0.65–0.88, *P* < 0.001). Participants with income over AUD600 per week consumed methamphetamine more frequently (on average 1.28 times more) than those with income below AUD600 and those who were employed consumed less frequently (on average 0.87 times less). Sensitivity analysis confirmed a near‐identical pattern for those who reported methamphetamine use after pandemic onset (Supporting information Table S3).

**TABLE 5:** Mixed effects model showing associations frequencies of use for methamphetamine, alcohol, tobacco and other illicit drugs: model estimates, standard error values and probability values (*P* values) (*n* = 277 participants).

Covariates	Methamphetamine use frequency			Alcohol use frequency			Tobacco use frequency			Other illicit drug use frequency		
	Relative risk (SE)	CI	*P*	Relative risk (SE)	CI	*P*	Relative risk (SE)	CI	*P*	Relative risk (SE)	CI	*P*
Age at baseline, years	1.00 (0.00)	0.99–1.01	0.739	0.98 (0.01)	0.96–1.00	0.091	1.00 (0.01)	0.99–1.01	0.885	0.98 (0.01)	0.97–1.00	0.068
Male	1.12 (0.11)	0.93–1.35	0.222	1.22 (0.26)	0.80–1.84	0.358	1.00 (0.10)	0.83–1.21	0.972	0.99 (0.16)	0.72–1.36	0.949
Employed	0.87 (0.06)	0.77–0.99	**0.035**	1.28 (0.11)	1.08–1.51	**0.005**	1.02 (0.04)	0.94–1.12	0.576	0.97 (0.09)	0.81–1.16	0.710
Income: above AUD600 per week	1.28 (0.07)	1.16–1.42	**<0.001**	1.15 (0.09)	0.99–1.33	0.077	0.97 (0.04)	0.90–1.04	0.394	0.91 (0.07)	0.78–1.06	0.235
Stable accommodation	0.91 (0.05)	0.81–1.02	0.095	0.96 (0.09)	0.79–1.15	0.637	0.98 (0.04)	0.90–1.07	0.700	1.07 (0.10)	0.89–1.30	0.455
Known drug source	1.01 (0.08)	0.87–1.18	0.859									
On a drug treatment programme in the past 12 months	1.02 (0.05)	0.92–1.12	0.761									
Time in the study	0.95 (0.02)	0.91–0.99	**0.013**	1.07 (0.04)	0.99–1.15	0.078	1.10 (0.02)	1.07–1.14	**<0.001**	1.06 (0.04)	0.99–1.13	0.113
Melbourne recruitment	0.76 (0.06)	0.65–0.88	**<0.001**	1.24 (0.14)	1.00–1.54	**0.052**	0.96 (0.05)	0.86–1.07	0.491	0.77 (0.09)	0.61–0.97	**0.027**
COVID‐19 restriction period (Ref: Pre‐COVID‐19)			**0.003**			0.964			**<0.001**			**0.027**
During Lockdown 1	0.46 (0.13)	0.27–0.79	**0.005**	1.00 (0.12)	0.79–1.28	0.974	0.94 (0.06)	0.83–1.06	0.319	1.12 (0.23)	0.75–1.67	0.578
Between Lockdowns 1 and 2	0.91 (0.18)	0.61–1.34	0.627	1.00 (0.14)	0.76–1.32	0.985	0.88 (0.06)	0.78–1.01	0.060	1.55 (0.37)	0.98–2.46	0.061
During Lockdown 2	0.76 (0.08)	0.62–0.92	**0.006**	0.93 (0.10)	0.76–1.15	0.511	0.86 (0.04)	0.77–0.95	**0.003**	1.06 (0.15)	0.81–1.40	0.661
After Lockdown 2	0.70 (0.08)	0.56–0.86	**0.001**	0.95 (0.11)	0.76–1.19	0.672	0.78 (0.04)	0.70–0.87	**<0.001**	0.71 (0.11)	0.52–0.95	**0.024**
Interaction between COVID‐19 restriction period and recruitment location (Ref: Pre‐COVID ‐19 and regional Victoria)			**0.054**									0.364
During Lockdown 1: Melbourne	1.51 (0.52)	0.77–2.98	0.229							0.88 (0.23)	0.53–1.46	0.624
Between Lockdowns 1 and 2: Melbourne	1.19 (0.27)	0.76–1.85	0.445							0.59 (0.17)	0.34–1.04	0.068
During Lockdown 2: Melbourne	1.26 (0.16)	0.98–1.63	0.073							0.87 (0.16)	0.61–1.24	0.442
After Lockdown 2: Melbourne	1.37 (0.18)	1.05–1.78	**0.019**							1.07 (0.20)	0.74–1.54	0.724
(Intercept)	2.90 (0.35)	2.29–3.67	**<0.001**	0.30 (0.06)	0.20–0.46	**<0.001**	3.82 (0.36)	3.19–4.59	**<0.001**	1.96 (0.33)	1.40–2.74	**<0.001**

*Note*: Statistical significances are provided in bold font.

Abbreviations: COVID‐19, coronavirus 2019; Ref, reference category.

### Frequency of other drug use

Table 5 shows the relationships between the covariates and use frequency of alcohol, tobacco and other illicit drugs. There was no effect of restriction period on the use frequencies of alcohol. Employed participants consumed alcohol more frequently than unemployed participants by 28% (95% CI = 8% to 51%, *P* = 0.005). Compared to the pre–COVID‐19 period, tobacco use frequency decreased significantly during Lockdown 2 and after Lockdown 2 by a factor of 14% and 22%, respectively. Tobacco use frequency was more likely to increase with time in the study by 10% in each year. Other illicit drugs use frequency decreased significantly after Lockdown 2 by 29% (RR = 0.71, 95% CI = 0.52–0.95, *P* = 0.024) and 24% (RR = 0.76, 95% CI = 0.56–1.02, *P* = 0.065) compared to pre‐COVID‐19 figures in regional Victoria and Melbourne, respectively. Use frequencies in Melbourne during Lockdown 2 were significantly less than regional Victoria by 33% (RR = 0.67, 95% CI = 0.46–0.97, *P* = 0.034).

## DISCUSSION

COVID‐19 led to public health measures including unprecedented border controls and restrictions on movement and gathering in Victoria, Australia, with its capital cited as the world’s most locked‐down city by the end of 2021 [14]. We found strong evidence that these measures were related to higher methamphetamine prices and reduced use, with only weak evidence that other drug use increased, corroborating previous cross‐sectional studies of people who use drugs [7, 19]. These effects were most marked during Lockdown 2, when the most severe restrictions on movement in Melbourne were applied. Unexpectedly, however, price increases were larger in regional Victoria, where restrictions on movement during Lockdown 2 were less stringent (no curfew, no five kilometre limit on movement and no one hour limit on exercise). In hindsight, this effect probably reflects the so‐called ‘ring of steel’ around Melbourne—a hard border enforced by numerous police on all roads out. This, coupled with similar intense enforcement of adjacent state borders, likely restricted the movement of illicit drug supply to regional Victoria and further increased prices. Prices did not return to pre‐COVID‐19 levels after harder restrictions were lifted and remained significantly higher than pre‐COVID‐19 levels. Similarly, methamphetamine and other drug use frequency did not return to pre‐pandemic levels within the timeframe we considered. Our findings contrast with those from a recent study of people who use drugs [7], which showed no effects of COVID‐19 restrictions on illicit drug prices, but these findings from serial cross‐sectional samples may reflect sampling biases because that study transitioned from face‐to‐face to telephone interviews, the inclusion of parts of Australia that had few restrictions on movement after the initial nationwide lockdown (Lockdown 1) and the inclusion of people who typically use ecstasy rather than methamphetamine.

Market changes related to COVID‐19 restrictions and the restrictions themselves were expected to affect participants’ drug use behaviours. We found reduced methamphetamine use frequency among participants in regional Victoria, again possibly because of changes in supply resulting from intense border controls around Melbourne. The absence of change in Melbourne was unexpected, particularly given evidence of reductions in use in cross‐sectional samples [7], but highlights how patterns of drug use can persist despite major impacts from ‘big events’ [20]. These changes in methamphetamine use were not offset by changes in use of other illicit drugs or legally available drugs such as alcohol or tobacco; indeed, tobacco use declined during lockdowns. We found few other effects of fixed or time‐varying factors, employed participants reporting less methamphetamine and more alcohol consumption and those with weekly income above AUD600 reporting higher methamphetamine use frequencies. Although we found no significant effects of past‐year drug treatment engagement on methamphetamine use frequency, drug treatment or ongoing support may provide opportunities to consolidate reductions in use related to ‘big events’ and the pandemic response accelerated remote and virtual engagement modalities in Australia, also presenting an opportunity for the promotion of these services [2]. Importantly, there was no evidence of time‐varying changes in drug source, suggesting market access continuity despite intense public health measures. The increase in income mirrored the pattern of methamphetamine price changes, except that the income distribution pattern returned to pre‐pandemic levels at the end the study period, suggesting that these market impacts occurred independently of changes in pandemic‐related income support [22].

Ours is one of few longitudinal studies of drug markets and substance use during COVID‐19 pandemic, anchored with observations before its onset. Our study is limited by small numbers of interviews during some restriction periods such as ‘during Lockdown 1’ and ‘between Lockdowns 1 and 2’ because of the short length of those restriction periods. This limits the statistical power available for analyses of some phases of the pandemic. As we focused on the impacts during the initial stages of the pandemic, periods with the most intense restrictions and the time periods immediately after, we measured only what are relatively short‐term impacts of the pandemic. Further work is needed to determine what, if any, changes result from the pandemic in the longer term. Although within‐person changes in drug use patterns and behaviours are a powerful tool for examining these impacts, recruitment biases remain, with our post‐COVID‐19 sample more likely to include residents of Melbourne than regional Victoria. Further, our time‐varying exposure of drug treatment use was captured over the previous 12 months, meaning that it was not linked directly to implementation of public health measures related to COVID‐19. Our study is also reliant on self‐report, but such data have proved reliable in similar previous work [23].

Our study highlights how COVID‐19 public health measures in Victoria, Australia, were associated with profound changes in the methamphetamine market. Methamphetamine prices increased, most markedly in regional Victoria, where methamphetamine use frequency among the cohort decreased. Despite a decline in prices after measures were relaxed, these changes in methamphetamine use frequency were sustained and did not appear to be offset by switches to other drugs, illicit or licit. These findings highlight how environmental shocks can be associated with profound effects on drug use behaviours [24], with important lessons for understanding future shocks and pandemic preparedness. Further work is required to determine how these changes related to drug‐related harms and the use of support services such as drug treatment.

## AUTHOR CONTRIBUTIONS

Kasun Rathnayake, Paul A. Agius and Paul Dietze conceived and designed the study. Kasun Rathnayake extracted the data from the database and prepared data for the analysis. Kasun Rathnayake performed the statistical analysis, supported by Paul A. Agius and supervised by Anna Wilkinson, and Paul Dietze. Paul Dietze drafted the manuscript, supported by Kasun Rathnayake. Bernadette Ward, Matthew Hickman, Lisa Maher, Mark Stoove, Joseph Doyle, Margaret Hellard, Brendan Quinn, SIone Crawford and Keith Sutton: Writing – review & editing‐equal. All authors critically revised the manuscript for intellectual content. Paul Dietze supervised the work. All authors read and gave final approval of this manuscript to be submitted. Paul Dietze had final responsibility for the decision to submit for publication.

## DECLARATION OF INTERESTS

P.D., M.S. and L.M. are funded by National Health and Medical Research Council (NHMRC) Senior Research Fellowships, M. Hellard by an NHMRC L3 Investigator Grant. P.D. has received funding from Gilead Sciences and Indivior for work unrelated to this study and was an unpaid member of an advisory board for an intranasal naloxone product. P.D. and M.S. have received investigator‐driven research funding from Gilead Sciences for work on hepatitis C unrelated to this work. M.S has received funding from AbbVie for work on hepatitis C unrelated to this work. M. Hickman acknowledges funding from NIHR Health Protection Research Unit in Behavioural Science and Evaluation. M. Hickman has received unrestricted and unrelated speaker fees and travel expenses in the past 3 years from Gilead and MSD. J.D., M. Hellard and M.S. receive investigator‐initiated research funding support from Gilead Sciences, AbbVie, Bristol Myers Squibb and Merck. J.D. and his institution have received consultancy fees from Gilead, AbbVie and Merck. Burnet Institute receives infrastructure support from Victorian Government, Australia.

## Supporting information


**Table S1** COVID‐19 restrictions for different time periods for Melbourne and regional Victoria.
**Table S2** Table of variable descriptions.
**Table S3** Sensitivity analysis based on the mixed effects model showing associations with methamphetamine, alcohol, tobacco, and other illicit drugs use frequency: Model estimates, standard error values, and probability values (p‐values) (*n* = 215 participants).
**Table S4** Linear mixed effects model showing associations with methamphetamine price paid per gram: Model estimates, effect sizes (*Cohen*’*s D (d)*) and probability values (*P*‐values) (*n* = 185 participants).
**Table S5** Mixed effects model showing associations frequencies of use for methamphetamine, alcohol, tobacco, and other illicit drugs: Model estimates, effect sizes (Cohen’s D (*d*)), and probability values (*P*‐values) (*n* = 277 participants).

## Data Availability

The data that support the findings of this study are available from the corresponding author on reasonable request.
