# Supplementary material for: The impacts of COVID‐19 measures on drug markets and drug use among a cohort of people who use methamphetamine in Victoria, Australia
Source: Addiction. 2023 Apr 2;118(8):1557–68. doi: 10.1111/add.16189 (PMC10953406; doi:10.1111/add.16189)
Supplement: Supplementary file 1 — Table S1 COVID‐19 restrictions for different time periods for Melbourne and regional Victoria. Table S2 Table of variable descriptions. Table S3 Sensitivity analysis based on the mixed effects model showing associations with methamphetamine, alcohol, tobacco, and other illicit drugs use frequency: Model estimates, standard error values, and probability values (p‐values) (n = 215 participants). Table S4 Linear mixed effects model showing associations with methamphetamine price paid per gram: Model estimates, effect sizes (Cohen’s D (d)) and probability values (P‐values) (n = 185 participants). Table S5 Mixed effects model showing associations frequencies of use for methamphetamine, alcohol, tobacco, and other illicit drugs: Model estimates, effect sizes (Cohen’s D (d)), and probability values (P‐values) (n = 277 participants). [file ADD-118-1557-s001.docx]

**SUPPLEMENTARY MATERIALS:**

|  | **Restrictions – Melbourne** | **Restrictions – Regional Victoria** |
| --- | --- | --- |
| **Lockdown 1**  From 16/03/2020 to 12/05/2020 | - Suspended non-essential gatherings more than 100 people - Closed pubs, clubs, cinemas, entertainment venues, casinos, and night clubs - Two weeks mandatory quarantine for people arriving from overseas - Victorians are allowed to leave the house for four reasons: essential shopping for food and medical supplies, caregiving, essential work, and education, exercising - Restrictions on inside home (5 people) and outdoor (20 people) gatherings | - Suspended non-essential gatherings more than 100 people - Closed pubs, clubs, cinemas, entertainment venues, casinos, and night clubs - Two weeks mandatory quarantine for people arriving from overseas - Victorians are allowed to leave the house for four reasons: essential shopping for food and medical supplies, caregiving, essential work, and education, exercising - Restrictions on inside home (5 people) and outdoor (20 people) gatherings |
| **Between Lockdowns 1 & 2**  From 13/05/2020 to 30/06/2020 | - Allowed private and public gatherings up to 20 visitors - Restaurants, cafes, and pubs are open for 20 seated patrons - Reduced private gatherings limit back to five visitors | - Allowed private and public gatherings up to 20 visitors - Restaurants, cafes, and pubs are open for 20 seated patrons - Reduced private gatherings limit back to five visitors |
| **Lockdown 2**  From 01/07/2020 to 28/10/2020 | - Stage 3^*^ restrictions in place in Melbourne on 08/07/2020 - 9 public housing estates moved to “hard lockdown” - Stage 4^#^ restrictions in place in Melbourne on 02/08/2020 - Stage 4 restrictions relaxed on 27/10/2020 including allowing public and private gatherings (limits apply) and open up hospitality venues, but can only travel up to 25km from home | - Stage 3^*^ restrictions in place in regional Victoria on 05/08/2020 - Restrictions to enter or leave Melbourne or Mitchell Shire (adjacent to the north of Melbourne) - Stage restrictions relaxed on 16/09/2020, but cannot travel to Melbourne |
| **After Lockdown 2**  From 29/10/2020 to 25/05/2021 | - 25km travel restriction removed and intrastate travel allowed on 08/11/2020 - COVID Safe summer introduced on 06/12/2020 including public gatherings up to 100 people and 30 visitors per home in a day - 5-day circuit breaker restrictions introduced on 12/02/2021 including only four reasons to leave home within 5km of home - Restrictions further relaxed including 100 people at home gatherings each day and 200 people outdoors - Restriction introduced to greater Melbourne on 25/05/2021 including public gatherings up to 30 people and five visitors per home in a day | - 25km travel restriction removed and intrastate travel allowed on 08/11/2020 - COVID Safe summer introduced on 06/12/2020 including public gatherings up to 100 people and 30 visitors per home in a day - 5-day circuit breaker restrictions introduced on 12/02/2021 including only four reasons to leave home within 5km of home - Restrictions further relaxed including 100 people at home gatherings each day and 200 people outdoors |

**Supplementary Table 1: COVID-19 restrictions for different time periods for Melbourne and regional Victoria**

^*^Stage 3 restrictions:

- Can leave home only for four reasons: essential shopping for food and medical supplies, caregiving, essential work, and education, exercising with one other person or household members
- Cafes are restaurants are open only for delivery and take-away
- Pubs, clubs, bars, and nightclubs are closed
- Beauty and personal services are closed
- Entertainment and cultural venues are closed
- Community sports are stopped

^#^Stage 4 restrictions:

In addition to the Stage 3 restrictions, the following restrictions were imposed:

- Curfew from 8pm to 5am
- Restrictions on travel up to 5km from home
- Exercise restricted to one hour outside a day with one other person
- One person per household can leave home for essential goods and service

**Supplementary Table 2: Table of variable descriptions**

|  | Categories | Description |
| --- | --- | --- |
| Age at baseline | Numerical variable | Age of the participant at the enrolment to the study (in years) |
| Gender | Ref**:** Female  Male | Gender of the participant |
| Employment status | Ref**:** Unemployed (retired, home duty, volunteer)  Employed (work as a full-time or part-time worker, self-employer, on job keeper, cash in hand jobs) | Employment status at each of the interview |
| Accommodation stability | Ref**:** Unstable (living in a caravan, friend’s place, house sitting)  Stable (living in a community housing, owned property, share house) | Place of living at the time of the interview |
| Location | Ref**:** Regional Victoria (Shepparton, Bendigo and LaTrobe Valley)  Melbourne (Metropolitan Melbourne) | Recruitment location of the participant |
| Income level per week | Ref**:** Below AUD600  Above AUD600 | Average weekly income (before tax) in the past six months from all sources |
| Drug source status | Ref**:** Unknown (Purchased from online, dark web, craigslist)  Known (Purchased from a relative, a family member, a partner) | Method of sourcing methamphetamine |
| Drug treatment status | Ref**:** No  Yes (Drug treatment programs such as individual drug counselling, group counselling, residential detoxification, outpatient detoxification, residential rehabilitation, pharmacotherapy treatment) | Status of engaging any of the drug treatment programs related to their methamphetamine use in the previous 12 months |
| Time in the study | Numerical variable | Time in the study since enrolment (in years) |

**Supplementary Table 3: Sensitivity analysis based on the mixed effects model showing associations with methamphetamine, alcohol, tobacco, and other illicit drugs use frequency: Model estimates, standard error values, and probability values (p-values) (n=215 participants)**

|  | **Methamphetamine Use Frequency** | | | **Alcohol Use Frequency** | | | **Tobacco Use Frequency** | | | **Other illicit drug Use Frequency** | | |
| --- | --- | --- | --- | --- | --- | --- | --- | --- | --- | --- | --- | --- |
| *Predictors* | *Relative Risk*  *(se)* | *CI* | *p* | *Relative Risk*  *(se)* | *CI* | *p* | *Relative Risk*  *(se)* | *CI* | *p* | *Relative Risk*  *(se)* | *CI* | *p* |
| Age at baseline, Years | 0.99 (0.01) | 0.98 – 1.00 | 0.239 | 0.98 (0.01) | 0.95 – 1.00 | 0.063 | 1.00 (0.01) | 0.99 – 1.01 | 0.764 | 0.99 (0.01) | 0.97 – 1.01 | 0.278 |
| Male | 1.18 (0.12) | 0.97 – 1.44 | 0.090 | 1.11 (0.27) | 0.69 – 1.79 | 0.656 | 0.94 (0.10) | 0.76 – 1.16 | 0.570 | 0.97 (0.18) | 0.68 – 1.39 | 0.873 |
| Employed | 0.94 (0.06) | 0.82 – 1.07 | 0.347 | 1.30 (0.13) | 1.07 – 1.58 | **0.009** | 1.07 (0.05) | 0.97 – 1.18 | 0.296 | 0.96 (0.10) | 0.78 – 1.18 | 0.701 |
| Earn above AUD600 | 1.22 (0.07) | 1.09 – 1.37 | **<0.001** | 1.16 (0.10) | 0.97 – 1.38 | 0.108 | 0.95 (0.04) | 0.87 – 1.03 | 0.214 | 0.86 (0.08) | 0.72 – 1.03 | 0.097 |
| Stable accommodation | 0.94 (0.06) | 0.83 – 1.07 | 0.370 | 0.96 (0.10) | 0.78 – 1.18 | 0.716 | 1.00 (0.05) | 0.91 – 1.10 | 0.987 | 1.08 (0.11) | 0.88 – 1.32 | 0.480 |
| Known drug source | 1.03 (0.09) | 0.87 – 1.21 | 0.733 |  |  |  |  |  |  |  |  |  |
| On a drug treatment program in the past 12 months | 0.97 (0.05) | 0.87 – 1.09 | 0.636 |  |  |  |  |  |  |  |  |  |
| Time in the study | 0.96 (0.02) | 0.92 – 1.00 | 0.081 | 1.07 (0.05) | 0.98 – 1.17 | 0.132 | 1.15 (0.02) | 1.10 – 1.19 | **<0.001** | 1.05 (0.04) | 0.97 – 1.13 | 0.275 |
| Melbourne recruitment | 0.77 (0.06) | 0.66 – 0.91 | **0.002** | 1.12 (0.16) | 0.85 – 1.47 | 0.409 | 0.96 (0.06) | 0.85 – 1.09 | 0.561 | 0.70 (0.10) | 0.53 – 0.93 | **0.012** |
| **COVID-19 restriction period (Ref: Pre-COVID-19)** |  |  | **0.043** |  |  | 0.940 |  |  | **<0.001** |  |  | **0.121** |
| During Lockdown 1 | 0.46 (0.13) | 0.27 – 0.80 | **0.006** | 0.92 (0.13) | 0.70 – 1.20 | 0.536 | 0.87 (0.06) | 0.76 – 0.99 | **0.035** | 0.97 (0.21) | 0.63 – 1.47 | 0.873 |
| Between Lockdowns 1 & 2 | 0.88 (0.18) | 0.60 – 1.31 | 0.533 | 0.90 (0.14) | 0.66 – 1.22 | 0.502 | 0.86 (0.06) | 0.75 – 0.99 | **0.038** | 1.60 (0.39) | 0.99 – 2.59 | 0.054 |
| During Lockdown 2 | 0.76 (0.08) | 0.63 – 0.93 | **0.008** | 0.94 (0.11) | 0.74 – 1.19 | 0.611 | 0.81 (0.05) | 0.73 – 0.91 | **<0.001** | 1.08 (0.16) | 0.81 – 1.44 | 0.615 |
| After Lockdown 2 | 0.73 (0.08) | 0.59 – 0.91 | **0.004** | 0.97 (0.13) | 0.75 – 1.26 | 0.834 | 0.75 (0.05) | 0.67 – 0.85 | **<0.001** | 0.73 (0.12) | 0.52 – 1.02 | 0.062 |
| **Interaction between COVID-19 restriction period and recruitment location (Ref: Pre-COVID-19 and regional Victoria)** |  |  | **0.014** |  |  |  |  |  |  |  |  | **0.441** |
| During Lockdown 1: Melbourne | 1.94 (0.67) | 0.98 – 3.83 | 0.057 |  |  |  |  |  |  | 1.07 (0.29) | 0.63 – 1.84 | 0.794 |
| Between Lockdowns 1 & 2: Melbourne | 1.30 (0.29) | 0.83 – 2.02 | 0.247 |  |  |  |  |  |  | 0.62 (0.18) | 0.35 – 1.11 | 0.109 |
| During Lockdown 2: Melbourne | 1.25 (0.16) | 0.97 – 1.61 | 0.085 |  |  |  |  |  |  | 0.99 (0.19) | 0.68 – 1.43 | 0.943 |
| After Lockdown 2: Melbourne | 1.41 (0.19) | 1.09 – 1.84 | **0.010** |  |  |  |  |  |  | 1.20 (0.25) | 0.80 – 1.81 | 0.373 |
| (Intercept) | 2.86 (0.37) | 2.21 – 3.69 | **<0.001** | 0.31 (0.07) | 0.19 – 0.49 | **<0.001** | 3.88 (0.39) | 3.18 – 4.73 | **<0.001** | 2.18 (0.41) | 1.51 – 3.14 | **<0.001** |

**Supplementary Table 4: Linear mixed effects model showing associations with methamphetamine price paid per gram: Model estimates, effect sizes (*Cohen’s D (d)*) and probability values (p-values) (n=185 participants)**

|  | **Price paid per gram** | | |
| --- | --- | --- | --- |
| *Covariates* | *Estimate (d)* | *CI* | *p* |
| Age at baseline, years | 1.61 (0.12) | -0.32 – 3.53 | 0.102 |
| Male | -32.79 (-0.20) | -68.56 – 2.98 | 0.072 |
| Employed | 20.19 (0.12) | -16.32 – 56.70 | 0.278 |
| Income above AUD600 per week | -12.12 (-0.07) | -44.80 – 20.56 | 0.467 |
| Stable accommodation | -2.87 (-0.02) | -39.30 – 33.56 | 0.877 |
| Known drug source | 8.52 (0.05) | -42.97 – 60.02 | 0.745 |
| On a drug treatment program in the past 12 months | 14.55 (0.09) | -16.36 – 45.46 | 0.356 |
| Time in the study | -32.27 (0.98) | -45.15 – -19.38 | **<0.001** |
| Melbourne recruitment | -29.14 (-0.35) | -66.75 – 8.47 | 0.129 |
| **COVID-19 restriction period (Ref: Pre-COVID-19)** |  |  | **<0.001** |
| During lockdown 1 | 7.74 (0.04) | -124.91 – 140.40 | 0.909 |
| Between lockdowns 1 & 2 | 158.20 (0.96) | 68.15 – 248.25 | **0.001** |
| During lockdown 2 | 456.51 (2.48) | 402.71 – 510.32 | **<0.001** |
| After lockdown 2 | 263.68 (1.52) | 203.21 – 324.15 | **<0.001** |
| **Interaction between COVID-19 restriction period and recruitment location (Ref: Pre-COVID-19 and regional Victoria)** |  |  | 0.063 |
| During lockdown 1: Melbourne | -3.00 (0.02) | -178.06 – 172.05 | 0.973 |
| Between lockdowns 1 & 2: Melbourne | -2.22 (0.02) | -111.61 – 107.17 | 0.968 |
| During lockdown 2: Melbourne | -104.88 (0.89) | -174.37 – -35.39 | **0.003** |
| After lockdown 2: Melbourne | -30.84 (0.12) | -108.53 – 46.84 | 0.436 |
| (Intercept) | 395.55 (0.98) | 329.91 – 461.18 | **<0.001** |

**Supplementary Table 5: Mixed effects model showing associations frequencies of use for methamphetamine, alcohol, tobacco, and other illicit drugs: Model estimates, effect sizes (Cohen’s D (*d*)), and probability values (p-values) (n=277 participants)**

|  | **Methamphetamine use frequency** | | | **Alcohol use frequency** | | | **Tobacco use frequency** | | | **Other illicit drug use frequency** | | |
| --- | --- | --- | --- | --- | --- | --- | --- | --- | --- | --- | --- | --- |
| *Covariates* | *Relative risk*  *(d)* | *CI* | *p* | *Relative risk*  *(d)* | *CI* | *p* | *Relative risk*  *(d)* | *CI* | *p* | *Relative risk*  *(d)* | *CI* | *p* |
| Age at baseline, years | 1.00 (0.01) | 0.99 – 1.01 | 0.739 | 0.98 (-0.23) | 0.96 – 1.00 | 0.091 | 1.00 (0.00) | 0.99 – 1.01 | 0.885 | 0.98 (-0.18) | 0.97 – 1.00 | 0.068 |
| Male | 1.12 (0.12) | 0.93 – 1.35 | 0.222 | 1.22 (0.20) | 0.80 – 1.84 | 0.358 | 1.00 (0.00) | 0.83 – 1.21 | 0.972 | 0.99 (0.00) | 0.72 – 1.36 | 0.949 |
| Employed | 0.87 (-0.14) | 0.77 – 0.99 | **0.035** | 1.28 (0.25) | 1.08 – 1.51 | **0.005** | 1.02 (0.02) | 0.94 – 1.12 | 0.576 | 0.97 (-0.01) | 0.81 – 1.16 | 0.710 |
| Income: above AUD600 per week | 1.28 (0.25) | 1.16 – 1.42 | **<0.001** | 1.15 (0.14) | 0.99 – 1.33 | 0.077 | 0.97 (-0.03) | 0.90 – 1.04 | 0.394 | 0.91 (-0.04) | 0.78 – 1.06 | 0.235 |
| Stable accommodation | 0.91 (-0.10) | 0.81 – 1.02 | 0.095 | 0.96 (-0.05) | 0.79 – 1.15 | 0.637 | 0.98 (-0.02) | 0.90 – 1.07 | 0.7000 | 1.07 (0.03) | 0.89 – 1.30 | 0.455 |
| Known drug source | 1.01 (0.01) | 0.87 – 1.18 | 0.859 |  |  |  |  |  |  |  |  |  |
| On a drug treatment program in the past 12 months | 1.02 (0.02) | 0.92 – 1.12 | 0.761 |  |  |  |  |  |  |  |  |  |
| Time in the study | 0.95 (0.09) | 0.91 – 0.99 | **0.013** | 1.07 (0.12) | 0.99 – 1.15 | 0.078 | 1.10 (0.23) | 1.07 – 1.14 | **<0.001** | 1.06 (0.12) | 0.99 – 1.13 | 0.113 |
| Melbourne recruitment | 0.76 (-0.05) | 0.65 – 0.88 | **<0.001** | 1.24 (0.22) | 1.00 – 1.54 | **0.052** | 0.96 (-0.04) | 0.86 – 1.07 | 0.491 | 0.77 (-0.18) | 0.61 – 0.97 | **0.027** |
| **COVID-19 restriction period (Ref: Pre-COVID-19)** |  |  | **0.003** |  |  | 0.964 |  |  | **<0.001** |  |  | **0.027** |
| During lockdown 1 | 0.46 (-0.56) | 0.27 – 0.79 | **0.005** | 1.00 (0.00) | 0.79 – 1.28 | 0.974 | 0.94 (-0.06) | 0.83 – 1.06 | 0.319 | 1.12 (0.02) | 0.75 – 1.67 | 0.578 |
| Between lockdowns 1 & 2 | 0.91 (-0.01) | 0.61 – 1.34 | 0.627 | 1.00 (0.00) | 0.76 – 1.32 | 0.985 | 0.88 (-0.12) | 0.78 – 1.01 | 0.060 | 1.55 (0.08) | 0.98 – 2.46 | 0.061 |
| During lockdown 2 | 0.76 (-0.16) | 0.62 – 0.92 | **0.006** | 0.93 (-0.07) | 0.76 – 1.15 | 0.511 | 0.86 (-0.15) | 0.77 – 0.95 | **0.003** | 1.06 (0.00) | 0.81 – 1.40 | 0.661 |
| After lockdown 2 | 0.70 (-0.21) | 0.56 – 0.86 | **0.001** | 0.95 (-0.05) | 0.76 – 1.19 | 0.672 | 0.78 (-0.25) | 0.70 – 0.87 | **<0.001** | 0.71 (-0.13) | 0.52 – 0.95 | **0.024** |
| **Interaction between COVID-19 restriction period and recruitment location (Ref: Pre-COVID-19 and regional Victoria)** |  |  | **0.054** |  |  |  |  |  |  |  |  | **0.364** |
| During lockdown 1: Melbourne | 1.51 (0.02) | 0.77 – 2.98 | 0.229 |  |  |  |  |  |  | 0.88 (-0.08) | 0.53 – 1.46 | 0.624 |
| Between lockdowns 1 & 2: Melbourne | 1.19 (0.01) | 0.76 – 1.85 | 0.445 |  |  |  |  |  |  | 0.59 (-0.19) | 0.34 – 1.04 | 0.068 |
| During lockdown 2: Melbourne | 1.26 (0.06) | 0.98 – 1.63 | 0.073 |  |  |  |  |  |  | 0.87 (-0.05) | 0.61 – 1.24 | 0.442 |
| After lockdown 2: Melbourne | 1.37 (0.22) | 1.05 – 1.78 | **0.019** |  |  |  |  |  |  | 1.07 (0.04) | 0.74 – 1.54 | 0.724 |
| (Intercept) | 2.90 (0.28) | 2.29 – 3.67 | **<0.001** | 0.30 (-0.21) | 0.20 – 0.46 | **<0.001** | 3.82 (0.32) | 3.19 – 4.59 | **<0.001** | 1.96 (0.23) | 1.40 – 2.74 | **<0.001** |

Note: Cohen’s D values were calculated as a measure of effect sizes for these non-normal GLM models. Calculations were based on ‘*eff_size()*’ function which is available in ‘*emmeans’* R package. These effect size calculations were based transformed values with respect to the relevant link function in each model.
